# A Case of Cholera in Which Chloroform Was Successfully Given Internally

**Published:** 1849-02

**Authors:** R. S. Strother

**Affiliations:** Bardstown, Kentucky


ART. VII.—*A Case of Cholera in which Chloroform was successfully given internally.* By R. S. STROTHER, M.D., of Bardstown, Kentucky.

On a recent trip to New Orleans, I had an opportunity of trying chloroform in cholera. The vessel on which I returned had fifteen or eighteen cases of the disease on board, of which seven terminated fatally. In the spasmodic stage large doses of opium, morphia, and musk were used, but without saving a solitary case.

The next case that offered itself was that of a negro man, who was seized with profuse diarrhea. This was checked by opiates, and for thirty-six hours he took no medicine. At the expiration of this time I was called in haste to see him. The diarrhea had returned; he was vomiting incessantly, and his cramps were violent, affecting the muscles of his arms, legs, and abdomen. He was in great agony, and apparently in a hopeless condition. I at once gave him a hundred drops of chloroform in a little sweetened water, the effect of which was to arrest the vomiting; he vomited but once after taking it, and in fifteen minutes every symptom of the disease had disappeared. His rapidly declining pulse returned, his extremities grew warm, and he remained in a half-intoxicated state, perfectly free from pain, and with pleasant sensations, for six hours, which interval I seized for the administration of other appropriate remedies. I left the patient convalescent, and apparently out of danger.

The other cases by being taken in time, were cured by the usual remedies. Had they advanced to the stage in which I found this man, I should have given chloroform, for the purpose of suspending the disease and gaining time. As an antispasmodic, it is unquestionably the most efficient article known to the physician, and, I am persuaded, not more dangerous than the preparations of opium.

Bardstown, Jan. 8, 1849.

